# The simple regularities in the dynamics of online news impact

**DOI:** 10.1007/s42001-021-00140-w

**Published:** 2021-09-12

**Authors:** Matúš Medo, Manuel S. Mariani, Linyuan Lü

**Affiliations:** 1 Institute of Fundamental and Frontier Sciences, University of Electronic Science and Technology of China, Chengdu, 610054 People’s Republic of China; 2 Department of Radiation Oncology, Inselspital, University Hospital of Bern, University of Bern, 3010 Bern, Switzerland; 3 Department of Physics, University of Fribourg, 1700 Fribourg, Switzerland; 4 URPP Social Networks, Universität Zürich, 8050 Zurich, Switzerland; 5 Complex Systems Lab, Beijing Computational Science Research Center, Beijing, 100193 People’s Republic of China; 6 Yangtze Delta Region Institute (Huzhou), University of Electronic Science and Technology of China, Huzhou, 313001 People’s Republic of China

**Keywords:** Online information, Dynamics of impact, Collective attention, Evolving networks

## Abstract

Online news can quickly reach and affect millions of people, yet we do not know yet whether there exist potential dynamical regularities that govern their impact on the public. We use data from two major news outlets, BBC and New York Times, where the number of user comments can be used as a proxy of news impact. We find that the impact dynamics of online news articles does not exhibit popularity patterns found in many other social and information systems. In particular, we find that a simple exponential distribution yields a better fit to the empirical news impact distributions than a power-law distribution. This observation is explained by the lack or limited influence of the otherwise omnipresent rich-get-richer mechanism in the analyzed data. The temporal dynamics of the news impact exhibits a universal exponential decay which allows us to collapse individual news trajectories into an elementary single curve. We also show how daily variations of user activity directly influence the dynamics of the article impact. Our findings challenge the universal applicability of popularity dynamics patterns found in other social contexts.

**Supplementary Information:**

The online version contains supplementary material available at 10.1007/s42001-021-00140-w.

## Introduction

Consider a major news, like the results of the presidential elections or the onset of a global epidemic outbreak. In the 80s, we would have discovered it through traditional print and broadcast media. Today, new media and online platforms have disrupted not only the way we discover and consume information, but also the way we form our opinions and attitudes about critical topics for our society like politics [1, 15], science [7, 24], and public health [25, 26]. Online newspapers and social media platforms are now the majour sources of information about events in the world [44] and provide us with rich data for the study of human attention [32, 52]. Despite the rise of social media, traditional newspapers and mainstream media are still important information sources with large audiences. The importance of mainstrean news sources can be illustrated by, for example, Facebook temporarily increasing their weight in the internal news ranking system in an attempt to respond to misinformation spreading following the U. S. presidential election.^1^

Most online newspapers allow users to directly comment on news articles [29], creating a “digital public sphere” where participation is free, recent events are publicly discussed, and comments are visible to everyone [42]. In such a complex information ecosystem, some news articles impact thousands of users who actively discuss and share them in online platforms,^2^ whereas many others remain little noticed. Therefore, understanding the dynamics of the impact of online news articles is vital not only because it deepens our understanding of how information spreads throughout modern societies, but also because it can potentially help to counteract negative side effects of new media like the spreading of misinformation [13, 49] and the amplification of ideological segregation [16].

The unprecedented availability of big data on human online activity has allowed us to uncover and model patterns of human behavior and cultural products’ popularity in diverse contexts [22, 33], revealing universal regularities in the dynamics of cultural products as diverse as scientific papers [50], books [53], and songs [8], among others. As for online news articles, previous research has unveiled factors that make an online news article more likely to become popular, including story topic [9], content emotion [5], perceived objectivity [30], and format [34]. Yet, we do not know yet whether there exist universal regularities that govern the dynamics of online news articles’ impact. Does the impact of online news articles follow similar patterns as the impact of other types of information items? Is article impact broadly distributed? Are there universal impact patterns for online news articles? How predictable is the dynamics of attention decay for online news articles? To address these questions, we analyze a novel dataset that contains commenting sections of 3087 articles from the British Broadcasting Corporation (BBC) and a dataset that contains commenting sections of 2801 articles from the New York Times (NYT).

Previous works have generated insights that generalize well across domains: popularity and impact typically follow heavy-tailed distributions, leading to the emergence of a small number of “hits” [3, 46] with disproportionate popularity. These successful outliers emerge from a combination of quality (often referred to as fitness) and social amplification mechanisms such as the rich-get-richer phenomenon [36]. These regularities in popularity dynamics have been found to govern the popularity and impact dynamics of cultural items as diverse as scientific papers [36, 50], websites [27], books [53], and patents [20, 21], among others.

Surprisingly, we find none of these regularities in the impact of online news. Differently from the widespread heavy-tailed distributions of popularity and impact in social systems, news impact in terms of the number of received comments) is exponentially distributed. Different categories of news have widely different average comment counts, yet their distributions can be collapsed onto a universal exponential distribution. The exponential impact distribution results from the absence or saturation of the widely-studied preferential attachment mechanism. In line with recent findings on the attention decay in science and technology [8, 20], the decay of individual news articles follows a universal exponential form. The impact dynamics of online news articles can be reproduced by a parsimonious model with article-level fitness and exponential aging [18]. Building on this model, we can predict the articles’ long-term impact based on early activity. We study the impact of natural daily variations of user activity on the dynamics of the article impact and formulate a generalized dynamical model which includes the overall level of user activity as an additional factor along with article fitness and an aging term.

Our findings contribute to the literature on popularity dynamics [17, 22, 27, 36, 50, 53] by demonstrating that there is a limit to the generality of widely-observed patterns and mechanisms (such as preferential attachment). While previous studies have emphasized the generality of observed patterns of popularity and impact [8], future research might put more emphasis on identifying violations of pervasive patterns and the causes behind the observed violations. Besides, as managing and influencing the spreading of online information is vital for online newspapers and social platforms, our models and methods can be used to inform decisions by newspaper editors and content creators.

## Results

### News impact is exponentially distributed

By writing comments, the users demonstrate a higher level of engagement compared to only reading the article [29, 30]. Importantly, comments are read also by users who do not actively comment, indicating that they play an important role in how a news article is perceived by the public [4]. The number of comments can be thus considered as a useful proxy for the article impact [47]. To study the distribution of article impact, we discard potential multiple comments from a single user on a given news, thus counting the number of unique users commenting on each article. When all comments are used instead, the results do not change qualitatively (see Supplementary Information, SI, for the results). We further benefit from the additional category information provided directly by the news outlets for all news; the most populated categories are football (BBC) and national (NYT); see Tab. S1 in the SI for details. Since comment counts strongly depend on the category of the news, we analyze news impact individually for each category.

How is the article impact distributed? Impact distributions for creative works are typically found to be heavy-tailed: this is the case for scientific papers [36], patents [48], and books [53], among others. Broad popularity distributions are also typically found for user-generated content in online systems [10]. Based on these findings, one might expect that the article impact too follows a heavy-tailed distribution. Surprisingly, we find instead that the distributions exhibit exponential tails for both BBC and NYT data. Using the exponential distribution^3^$P(c)\sim \exp (-\lambda \,c)$ for $c\ge c_{\text {min}}$ and following the methodology introduced in Ref. [11], we obtain estimates for the lower bound ${\hat{c}}_{\text {min}}$ and the scaling parameter $\hat{\lambda }$, together with the *p* value obtained through the Kolmogorov–Smirnov test (see "Methods" section for details).

**Fig. 1:**
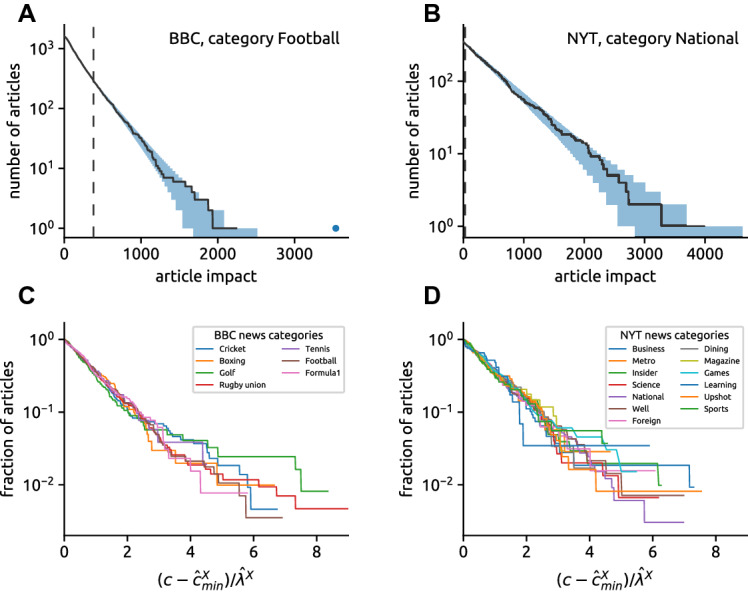
Article impact is exponentially distributed. **A**, **B** Comment count distributions for football news in the BBC data and national news in the NYT data. For the football category, the dot shows a single outlier that was identified in the exponential fitting. **C**, **D** The distribution of the transformed comment counts, $(c - {\hat{c}}_{\text {min}}^X) / \hat{\lambda }^X$, in individual news categories; here ${\hat{c}}_{\text {min}}^X$ and $\hat{\lambda }^X$ are the exponential distribution parameters for category *X*. Upon rescaling, a universal distribution of article impact emerges

For football BBC news, we find that the exponential tail of the distribution begins at ${\hat{c}}_{\text {min}}=381$ and comprises 284 articles (18% of all football articles). Ignoring a single outler with 3538 comments (see Sec. S2 in the SI for information on outlier detection), the estimated scaling parameter is $\hat{\lambda }=270\pm 17$ and the high *p* value of 0.96 indicates that the exponential distribution cannot be ruled out. The good fit can be visually appreciated by observing that the empirical distribution lies within the 5th–95th percentile range of synthetic exponentially distributed data generated with the estimated parameters (Fig. 1A). For national NYT news, the estimated lower bound is even lower, ${\hat{c}}_{\text {min}}=28$, and the *p* value is 0.53 (Fig. 1C) which again means that an exponential distribution is plausible. Detailed fitting results for all 20 news categories with at least 100 news are shown in the SI, Sec. S2. Importantly, the identified exponential tails are substantial, comprising more than 90% of news for 10 out of 20 analyzed news categories. The log-likelihood test [11] shows that an exponential distribution fits the data better than a power-law distribution for all categories but one (Learning in the NYT data, see Sec. S2.1 in the SI for additional information).

Inspired by the universality of scientific impact distributions [17, 39], we explore an intriguing possibility: By leveraging the estimated parameters, can we collapse the article impact distributions for different categories on top of each other? We find that this is the case: impact distributions in different categories collapse on top of each other after the comment counts are transformed as $(c - {\hat{c}}_{\text {min}}^X) /\hat{\lambda }^X$, where ${\hat{c}}_{\text {min}}^X$ and $\hat{\lambda }^X$ are the estimated lower bound and the scaling parameter for category *X*.

In summary, we find the impact distributions in individual news categories to be far from being power laws. Simple exponential fits work well in several categories where they describe the impact of a majority of news with remarkable veracity.

### The relation between article impact and user degree

The observed article impact distributions, albeit narrower than in many other technosocial systems, still comprise articles that have much greater audience than most other articles. This gives us the possibility to study the relation between impact of an article and degree of the users who have commented on this article. Such a relation can exist if, for example, little active users are mostly idle and comment only on high-impact articles; such a connection would in turn contribute to the high impact of those articles. To assess the level of degree assortativity in the bipartite article-user network, we divide both articles and users in five groups by their degree in such a way that the total degree in each group is approximately the same; groups 1 and 5 have the lowest and the highest degree users/articles, respectively. We then count the numbers of links between respective user and article groups and divide them with the average numbers of links observed in randomized networks. The resulting *link propensity* quantifies how much more likely (if propensity is above one) or less likely (if propensity is below one) are links between a given user and item group compared to a randomized network. For network randomization, we use the recently introduced Dynamic Configuration Model (DCM, [40]) which is a version of the classical configuration model for networks that grow in time. The DCM internally divides the network in *L* layers and *L* is a parameter of the model. Due to the quick aging that we observe in the analyzed datasets (see the following sections), we chose the number of layers to be the same as the number of days in each respective dataset (the results are robust with respect to the choice of *L*). See Ref. [37] for a principled way to determine *L* in a monopartite growing network.

**Fig. 2:**
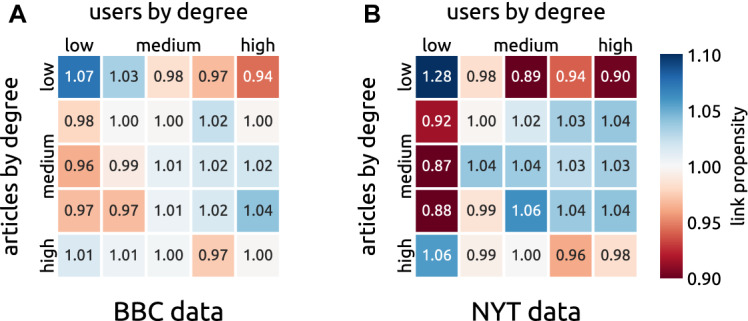
Relative link propensity between users and articles of various degree. Both users and articles are divided in five groups by their degree. The relative link propensity values quantify the excess (values greater than one) or lack (values smaller than one) of links between the respective pair of user and article groups as compared to randomized networks (we average over 1000 realizations of the DCM model [40]). The left and right panels show the results for the BBC data and the NYT data, respectively. While some statistically significant deviations from the null model can be observed (values that differ from one by more than, approximately, 0.02 have absolute *z*-scores above 3), only one of them (links between low-degree users and low-degree articles in the NYT data) is larger than 20% either way

Figure 2 shows that the relative link propensity is close to one for most pairs of user and article groups. One emerging pattern shared by the BBC and NYT data concerns the least popular articles which are commented by the least active users more than expected and by the most active users less than expected. By contrast, the original hypothesis of the most popular articles owing their popularity to little active users is ruled out by the results. For the BBC data, the relative link propensity between the most popular articles and the least active users does not differ significantly from one. For the NYT data, it is only 1.06 which means that the most popular articles do not receive 20% of their comments from the group of least active users who write 20% of all comments but $20\%\times 1.06\approx 21\%$ which is a negligible increase. We can conclude that the most popular articles receive comments from users of all degree values approximately in line with expectations.

### Preferential attachment plays a minor role in the dynamics of impact

The empirical exponential distributions of article impact inevitably lead us to investigate possible mechanisms behind their emergence. Motivated by existing results on the dynamics of impact for cultural products as diverse as scientific papers [36, 50], patents [20], and bestseller books [53], one expects two main forces shaping the dynamics of news impact [8]: preferential attachment and temporal decay. We start by addressing preferential attachment which implies that the rate at which article *i* receives new comments, $\varDelta c_i(t)/\varDelta t$ where $\varDelta c_i(t) = c_i(t + \varDelta t) - c_i(t)$, is a power-law function (most commonly, a linear function) of the number of already-received comments, $c_i(t)$.

**Fig. 3:**
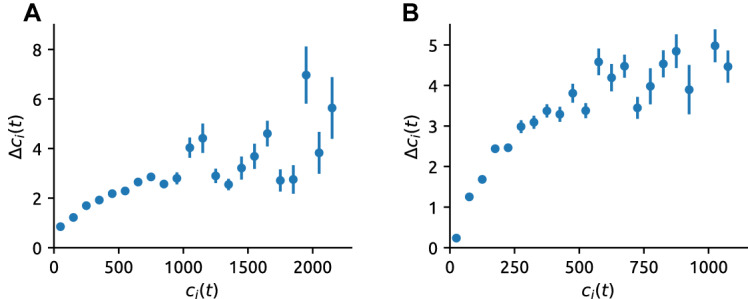
Preferential attachment in the dynamics of article impact. The number of new comments in $\varDelta t=10\,\text {min}$ as a function of the current number of comments. For the BBC data (**A**), the fit up to the comment count 800 yields the slowly-growing dependence proportional to $1+c_i(t)/220$. Above 800 comments, the dependence is even weaker (saturation). For the NYT data (**B**), sublinear preferential attachment with the exponent 0.79 is the best fit, followed by saturation for $c_i(t)\gtrsim 400$. Error bars indicate the standard error of the mean

In contrast with pervasive findings in the popularity dynamics literature, we find that preferential attachment is negligible in the BBC data (Fig. 3A) and exhibits clear sub-linearity and saturation for the NYT data (Fig. 3B). More specifically, in the BBC data, more than 200 comments are needed for an article to double its commenting rate with respect to a comment-free article. Furthermore, the observed weak growth of $\varDelta c_i(t)$ with $c_i(t)$ can be explained in terms of a dynamic model where no preferential attachment is present (see Fig. S9 in the SI). In the NYT data, $\varDelta c_i(t)$ first grows as a power of $c_i(t)$ with an exponent below one (sub-linear preferential attachment [28], see Fig. S10 in the SI) and becomes independent of $c_i(t)$ for $c_i(t)\gtrsim 400$. The lack of/saturation of preferential attachment has important consequences as it prevents a power-law degree distribution from emerging (see Section on modeling the article impact dynamics and Sec. S4 in the SI). In summary, we find that despite the articles’ comment counts explicitly reported by both BBC and NYT (see Fig. S1 in the SI), the impact of preferential attachment on the dynamics of news article impact is limited.

### The dynamics of article impact follows an exponential decay

**Fig. 4:**
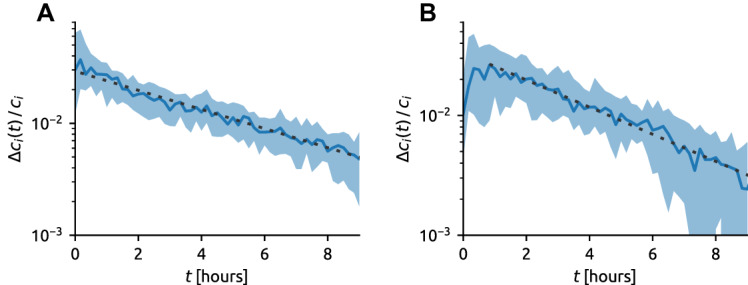
Aging in the dynamics of article impact. The number of new comments of an article, $\varDelta c_i(t)$, normalized by the final number of comments, $c_i$, as a function of its age, *t*, for the hit articles (90th percentile by the number of comments). The dotted lines indicate the linear fit for age 0–10 h; their slopes correspond to representative aging timescales $\varTheta =305\,\text {min}$ (BBC, panel ** A**) and $\varTheta =230\,\text {min}$ (NYT, panel ** B**), respectively. The time-of-day effects are suppressed here by including only the articles that appear in the morning between 9 am and noon (BBC) and between 2 pm and 5 pm GMT (NYT). The shaded areas there indicate the 20th–80th percentile range and the solid lines show the median values for the considered articles

Existing studies have found various functional forms for the decay of the impact of cultural items, including power-law [12], log-normal [50, 53], exponential [20, 38], stretched exponential [51], and biexponential [8]. To quantify the temporal decay of article impact, for each news *i*, we measure the news’ number of new comments relative to the article’s final comment count, $f_i(t):=\varDelta c_i(t)/c_i$, as a function of the article age, *t*. The normalization by the article’s final comment count makes the dynamics of articles of different ultimate impact directly comparable.^4^ For each age, *t*, we compute the median of $\varDelta c_i(t)/c_i$ over all considered articles, obtaining the representative decay function, *f*(*t*). We restrict the analysis to *hit articles* which, for the purpose of this work, are defined as the articles whose number of comments is above the 90th percentile (679 and 428 comments in BBC and NYT, respectively). To suppress the time-of-day effects, we include only the BBC articles that appeared in the morning between 9 am and noon—the 10 h range shown in Fig. 4A is thus a period when user activity is rather uniform at the BBC website. User activity is substantially lower in the night, which directly effects the evolution of $c_i(t)$ (see Sec. S3 in the SI for more details). For the same reasons, we focus on the NYT articles that appeared between 2 pm and 5 pm GMT.

We find that the articles’ temporal decay follows a universal exponential form (Fig. 4). In particular, the average decay function *f*(*t*) can be accurately fitted by an exponential function: $f(t)=\mathrm {e}^{-t/\varTheta }$ where $\varTheta =305\,\text {min}$ for BBC and $\varTheta =230\,\text {min}$ for NYT. While *f*(*t*) decreases exponentially in the BBC data during the whole observed range, it shows a short period (approximately 1 h) of increase in the NYT data. This is a direct consequence of the preferential attachment that applies for low comment counts—as the number of comments grows, the rate of commenting initially accelerates before aging in combination with sublinear/saturated preferential attachment eventually cause the rate of commenting to decrease.

Our finding of a regular exponential decay of article impact agrees with the report of real news on Twitter differing from rumors by exhibiting a regular pattern of a monotonous decrease of attention [31]. The observed exponential aging can be explained by a simple model where each article is of interest to a fixed pool of readers (the pool’s size is determined by the article’s attractiveness to the readers) and every reader has a fixed probability to write their comment (most readers comment in a discussion only once) per time unit [23]. The observed exponential decay can be also interpreted as a limit scenario of the bi-exponential impact decay predicted by a recent work based on a model with communication memory and cultural memory [8]. The reason why such a limit scenario holds for online news needs to be clarified by future research. A plausible hypothesis is that as the comments to online news articles unfold over a narrow time period following a news, we cannot use them to observe the process whereby the communication memory associated with an article is converted into cultural memory. If this is the case, the model in [8] predicts an exponential decay of collective attention, in line with our observed decay functions.

### Exponentially distributed fitness and exponential aging shape the dynamics of article impact

The impact dynamics for scientific papers [36, 50] and bestseller books [53] is typically modeled in terms of preferential attachment, fitness and aging. Building on these studies, a potential model for the commenting dynamics would assume that the expected rate at which article *i* receives new comments at time *t* is

1 $$\begin{aligned} \varDelta c_i(t)/\varDelta t = [1+c_i(t)]\,\eta _i\,f_i(t-t_i) \end{aligned}$$

where $1+c_i(t)$ is the preferential attachment factor, $\eta _i$ is the fitness factor, $f_i(t-t_i)$ denotes an article-dependent aging function, and $t_i$ is the appearance time of article *i*. In line with previous studies [36, 50], article fitness $\eta$ is a hidden intrinsic parameter that quantifies, other factors being equal, how a given article is attractive to the website’s audience. We refer to this model as the PFA model because it includes Preferential attachment, Fitness and Aging. In this model, a narrow exponential distribution of article fitness, $\rho (\eta )=\exp {(-\eta )}$, leads to the emergence of a power-law distribution of the comment count [36]. In other words, small differences in items’ fitness are amplified by preferential attachment and produce wide impact inequalities.

**Fig. 5:**
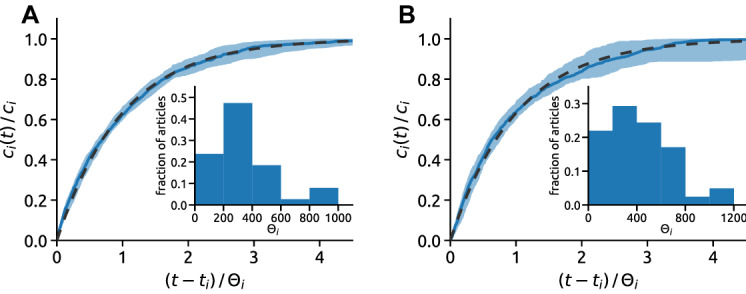
The universal dynamics of article impact. The comment count evolution in terms of the normalized article age $(t-t_i)/\Theta _i$ for the BBC (**A**) and NYT (**B**) data. The shaded areas there indicate the 20th–80th percentile range and the solid lines show the median values for the considered articles. The dashed line represents the proposed model and its solution given by Eq. (3). The inset shows the distribution of the timescales $\Theta _i$ obtained by minimizing the Kolmogorov–Smirnov statistic. Only “morning” articles are included in the analysis, as in Fig. 4

The observed weak preferential attachment and exponential temporal decay suggest a simpler model of the dynamics of article impact where only article fitness and exponential aging play a role. We thus assume that the rate at which article *i* receives new comments at time *t* is

2 $$\begin{aligned} \varDelta c_i(t)/\varDelta t = \eta _i\,f_i(t-t_i) \end{aligned}$$

which we refer as the FA (Fitness-Aging) model [18]. To accurately represent the commenting dynamics, we introduce individual aging timescales $\Theta _i$ and the aging factor in the form $f_i(t-t_i)=\exp [-(t-t_i)/\Theta _i]$. The aging timescales $\Theta _i$ are estimated from the empirical data by minimizing the Kolmogorov–Smirnov statistic between the comment count dynamics in the model and in the empirical data (see Sec. S6 in the SI). If $\Theta _i\gg 1$, the expected final comment count under the FA model is directly proportional to the product of the article fitness and the aging timescale, $\overline{c_i}=\eta _i\Theta _i$ (see Sec. S5 in the SI). The model further implies that

3 $$\begin{aligned} \frac{\overline{c_i(t)}}{\overline{c_i}} = 1 - \exp \biggl (-\frac{t-t_i}{\Theta _i}\biggr ). \end{aligned}$$

Motivated by this result, we measure the dynamics of the comment count normalized by the final comment count. We find that Eq. (3) captures the empirical dynamics remarkably well (Fig. 5) and allows us to collapse all article trajectories onto a universal curve (Fig. 5). This result demonstrates that the fitness-aging model captures the two essential factors that govern the dynamics of news article impact, and it further confirms that preferential attachment plays a negligible role in the emergence of hit articles. Combined with insights on empirical user dynamics (see Fig. 2 and Sec. S3 in the SI), Eq. (2) can be also formulated in terms of a growing bipartite network where $\varDelta c_i(t)/\varDelta i$ is the rate at which article node *i* attracts new links.

Since $\overline{c_i}\sim \eta _i\Theta _i$, exponentially distributed $\eta \Theta$ leads to the emergence of an exponential comment count distribution in line with the empirical data. When the aging timescales vary relatively little among the articles, as is the case here, the distribution of article fitness alone is approximately exponential. Interestingly, an exponential distribution of $\eta \Theta$ (referred to as total relevance therein) was reported in Ref. [36] for scientific papers and an exponential distribution of $\eta$ (in a model without aging) was reported in Ref. [27] for pages of the world wide Web. To identify theoretical mechanisms behind this widespread emergence of exponentially distributed fitness of items remains an important future challenge.

### Early activity can be used to predict article impact

The regular dynamics demonstrated by Fig. 5 suggests that the early commenting activity and the final article impact are highly correlated. To verify this conjecture, we study a classification problem where we aim to predict whether an article will become a hit (i.e., if it will belong to the 90th percentile by the final impact). We classify an article as positive if it belongs to the 90th percentile by the number of comments that it has attracted over the first $\varDelta t$ minutes, and negative otherwise. We evaluate the classifier using precision and AUC which are both classical information retrieval metrics [35] that range from zero (the worst result) to one (the best result). We find that the proposed simple classifier exhibits high values of precision and AUC even when $\varDelta t$ is short: precision exceeds 0.6 after 5 min, for example (see Table 1 for full results).

The observed predictability is unsurprising given previous results on the correlation between early and late popularity of online content [10, 43, 45]. However, previous studies interpreted the early-stage predictability of the virality of online cascades as a possible manifestation of cumulative advantage [43]. This cannot be the case here for online news where we demonstrated that preferential attachment has a negligible effect. Taken together, our findings suggest a somewhat simpler scenario: the news that are highly attractive for the public tend to receive more connections throughout their whole lifetime than less attractive news. In this sense, the impact of online news might be seen as more “meritocratic” than that of content in systems with preferential attachment: The news with truly high fitness are those that eventually succeed, regardless of cumulative advantage effects.

**Table 1:** Classification precision and AUC for the hit articles

	BBC data		NYT data	
$\varDelta t$	*P*	AUC	*P*	AUC
1	0.33	0.69	0.31	0.62
2	0.51	0.81	0.51	0.75
5	0.60	0.87	0.64	0.89
10	0.63	0.89	0.73	0.92
60	0.69	0.92	0.81	0.96
240	0.77	0.94	0.83	0.98
1200	0.93	0.99	0.95	0.99

### Circadian patterns of user activity patterns shape the dynamics of news impact

**Fig. 6:**
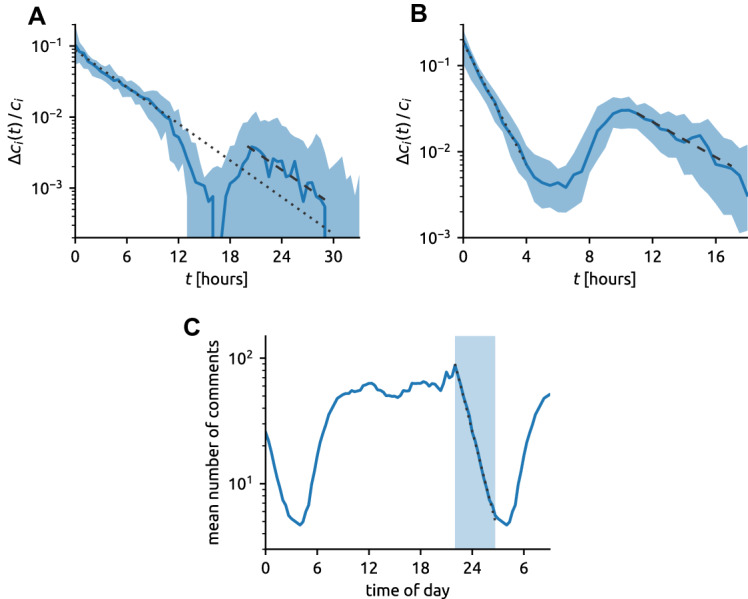
Interplay between exponential aging and circadian user activity patterns in the BBC data. ** A** The number of new comments of an article, $\varDelta c_i(t,\varDelta t)$, normalized by the final number of comments, $c_i$, as a function of its age, *t*, for morning hit articles (published between 9 am and noon). The dotted line indicates the linear fit for age 0–10 h (fitted timescale 302 min). The dashed line indicates the linear fit for age 20–29 h (fitted timescale 312 min). ** B** As (**A**) for evening articles (published between 9 pm and midnight). Timescales of the indicated fits are 74 min (first 4 h, dotted line) and 254 min (age 11–17 h, dashed line). In panels (**A**) and (**B**), the age bin size is 30 min to achieve better statistics for high article age.** C** The course of the mean number of comments in 20 min intervals during the day at the BBC Sport website. The fitted timescale of the exponential decrease between 10 pm and 3 am is 97 min

To study the impact dynamics, we have until now focused specifically on “morning” articles that benefit from high and approximately constant user activity for more than 10 h after their publication. We now turn our attention to the effect of overall user activity, which naturally decreases in the night (see Fig. S7 in the SI), on the dynamics of article impact. Figure 6A shows the evolution of the median number of new comments for the same set of morning articles over a longer period. This allows us to observe a decrease of commenting activity in the night (age 12–18 h) and renewed exponential decay on the second day (age 20–29 h) with the timescale of 312 min. The two fitted exponential decay timescales, 302 min for article age 0–10 h^5^ and 312 min for article age 20–29 h are remarkably close to each other. We see that after user activity recommences after a night, article aging continues in the same speed than before the night.

Figure 6B shows the evolution of the median number of new comments for evening hit articles (articles published between 9 pm and midnight). We see again two phases of exponential decay: an early phase during the night (age up to 4 h) with the timescale of 74 min and a late phase during the day (age 11–17 h) with the timescale 254 min. Albeit having a somewhat shorter timescale, the late phase is a direct equivalent of the previously observed aging of morning articles. We thus see that morning and evening articles exhibit similar aging during the day.

On the other hand, the early night aging of evening articles has a timescale which is significantly shorter than the aging timescle observed during the day. To better understand this fast aging, Fig. 6C shows the average overall activity on the BBC Sport website. We see that after an approximately constant activity during the day (from 8 am until 8 pm) and a small peak in the evening (from 8 pm until 10 pm) when many sport events take place, user activity dramatically decreases after 10 pm. Moreover, the user activity decay during the first 5 h of the decrease (from 10 pm until 3 am) is a nearly perfect exponential with the fitted timescale of 97 min. Varying user activity can be included in the previously introduced FA model described by Eq. (2) by introducing it as an additional multiplicative factor, thus obtaining an FAA (Fitness–Aging–Activity) model. The rate at which article *i* receives new comments at time *t* then reads

4 $$\begin{aligned} \varDelta c_i(t) / \varDelta t = \eta _i\,f_i(t-t_i)\,A(t) \end{aligned}$$

where $t_i$ and $\eta _i$ are the appearance time and fitness of article *i*, respectively, and *A*(*t*) is the overall activity factor which is common to all articles at the platform. If we now assume that article *i* appears when user activity, *A*(*t*), decreases exponentially, we obtain

$$\begin{aligned} \varDelta c_i(t) / \varDelta t = \eta _i\exp [-(t-t_i) / \Theta _A] A(t_i) \exp [-(t - t_i)/\Theta _U] \end{aligned}$$

where $\Theta _A$ and $\Theta _U$ are the article and user exponential decay timescales, respectively. The two exponential terms can be combined in one as

$$\begin{aligned} \exp [-(t-t_i) / \Theta _A] \times \exp [-(t - t_i)/\Theta _U] = \exp [-(t - t_i)/\Theta _J] \end{aligned}$$

where the joint exponential timescale, $\Theta _J$, has the form

5 $$\begin{aligned} 1/\Theta _J = 1/\Theta _A+1/\Theta _U. \end{aligned}$$

Using the fitted values $\Theta _A=302\,\text {min}$ and $\Theta _U=97\,\text {min}$, we obtain $\Theta _J=73\,\text {min}$ which is in an excellent agreement with the fitted aging timescale 74 min of evening articles during the night. Results are qualitatively similar for the NYT data (see Sec. S7 in the SI). We can thus conclude that the FAA model given by Eq. (4) presents an effective way of combining article dynamics with circadian and other patterns of varying user activity.

## Discussion

By analyzing data on the comments to online news articles in two major nationwide newspapers, we were able to uncover surprising empirical regularities that characterize the distribution of the impact of online news articles and the impact dynamics. In particular, we revealed two universal patterns: (1) For both newspapers, the distribution of the number of comments received by articles from various categories collapse onto a universal exponential curve and (2) the dynamics of the comment count of different news articles collapse onto a universal curve once appropriate rescaling is applied. The exponential impact distribution emerges from impact dynamics where preferential attachment plays a negligible role. This indicates that differently from other social systems [41, 51], popularity signals in online newspapers are not prominent enough to play a significant role. Main empirical dynamical patterns of article impact can be reproduced with a minimal model which combines article fitness, an aging term (which in our case has an exponential form), and overall user activity. When user activity is approximately constant, only article impact and aging remain and the resulting dynamics is particularly simple.

Our findings contrast with the previous literature on success and popularity that has emphasized that success and popularity are usually characterized by heavy-tailed distributions [6, 10, 27, 39], and that preferential attachment plays a key role in shaping the emergence of hits [27, 36, 50, 53]. Additional research is needed to quantify the relative importance of different factors that trigger user engagement in a news article (i.e., which article attributes contribute to its fitness [2, 5]), and how our findings generalize to different cultures and platforms in languages other than English.

We quantified the impact of a news article through the number of comments it received from the online newspaper’s readers. Other metrics of impact might be also relevant to news outlets. For example, the overall impact of a news can be quantified as a combination of the impact on the readers of the newspaper and the impact on users who shared or commented the news in different social media and news aggregation platforms. Uncovering the regularities of the news articles’ dynamics by incorporating data from social media and news aggregators is an important direction for future research, given the critical role of these platforms for news dissemination [14, 19].

Although our study focused on news outlets that only include verified news (BBC and NYT), our findings can inspire future studies related to the spreading of misinformation in online systems [13, 49]. Our results could serve as baselines in future studies that consider the commenting dynamics of both verified and false news. Do false news trigger different patterns of impact compared to true news? Is the diffusion of false and true news governed by different fundamental mechanisms? Understanding which mechanisms play a major role in engaging users and triggering their comments might suggest intervention strategies to prevent their impact.

The collected BBC data contain several other characteristics that have not been included in the present study: comment length, comment text, as well as the number of up-votes and down-votes for each comment. Their analysis can yield further patterns in article commenting. Of particular interest is the interplay between comment sentiment and the discussion activity is of particular interest. Do positive or controversial comments help fuel the discussion? Do early emotionally loaded comments influence the long-term tone of the discussion? Which factors contribute to users approval or disapproval of a comment? Such studies can help us understand how we discuss online and how to make these discussions more construcitve.

## Methods

### Empirical datatasets

We regularly crawled the sport section of the BBC website (its front page and the pages dedicated to individual sports) and collected the found news articles with commenting sections. From October 1, 2018 to June 30, 2019, we collected 3,087 articles that received 852,400 comments from 67,527 readers. Each article is assigned to a sport category. The most populated categories are Football (1590 articles), Rugby Union (439 articles), Cricket (240 articles), Tennis (162 articles), Formula 1 (139 articles), Golf (123 articles) and Boxing (103 articles). Each comment is time-stamped with the time resolution of 1 min. BBC typically closes commenting on the second midnight after the article has been published; most of them are therefore open for 24–48 h.

We complement the unique BBC dataset with a dataset containing articles with commenting sections from the New York Times (NYT).^6^ From January 1, 2017 to May 30, 2017, there are 2,801 articles that received 649,794 comments from 75,118 readers. Also here, each article is assigned to a category. Unlike for BBC, sport articles are a minority in the NYT data: The most populated categories are National (348 articles), Learning (306 articles), Magazine (262 articles), Sports (213 articles) and Foreign (204 articles). Each comment is time-stamped with the time resolution of 1 min. While some comments arrive long after the articles are published, the median time after which the hit articles (90th percentile by the comment count) receive 99% of their comments is less than 26 h. To study the article impact dynamics, we thus focus on the first 26 h of article age (the final article impact is nevertheless determined using all data). See Supplementary Information, Section S1, for detailed information about the datasets.

Both datasets can be represented as bipartite networks with user nodes and news article nodes. Each comment is then represented by a link between the user who wrote it and the news article to which the comment belongs. These networks evolve with time as the number of article nodes, user nodes, and links all gradually grow.

### Fitting the comment count distributions

The maximum likelihood estimate (MLE) of the scaling parameter of the exponential distribution is known to be the sample mean, $\hat{\lambda }= (\sum _{i=1}^n c_i) / n$. As can be seen from Fig. 1, the comment count distribution follows an exponential form starting from some lower bound ${\hat{c}}_{\text {min}}$. The MLE estimate then changes to $\hat{\lambda }({\hat{c}}_{\text {min}}) = [\sum _j (c_j - {\hat{c}}_{\text {min}})] / n({\hat{c}}_{\text {min}})$ where the summation is over *j* for which $c_j \ge {\hat{c}}_{\text {min}}$ and $n({\hat{c}}_{\text {min}}) = |\{j:\ c_j \ge {\hat{c}}_{\text {min}}\}|$ is the number of comment counts that match or exceed the lower bound. We assess the estimate uncertainty using non-parametric bootstrap—standard deviation of the MLE estimates is evaluated for 10,000 bootstrap realizations of the comment count data.

To determine ${\hat{c}}_{\text {min}}$, we follow the approach suggested by [11]: We choose ${\hat{c}}_{\text {min}}$ that minimizes the difference between the comment count distribution and the fitted exponential distribution as measured by the standard Kolmogorov–Smirnov statistic which has the form

6 $$\begin{aligned} D = \max _{c\ge {\hat{c}}_{\text {min}}} |S(c) - P(c)| \end{aligned}$$

where *S*(*c*) and *P*(*c*) are the cumulative distributions for the comment counts and the fitted exponential distribution, respectively. When the weighted Kolmogorov–Smirnov statistic [11] is used, which puts more weight on tails of the distributions, results do not change qualitatively. This further suggests that our fitting procedure and the conclusions drawn from the results are robust.

The next step is to test the hypothesis that the observed comment counts indeed follow an exponential distribution. We follow again [11] where the authors suggest to use the fitted parameters to generate synthetic exponentially distributed datasets, fit each of those datasets as described above, and finally calculate the *p* value as the fraction of synthetic datasets whose resulting *D* exceeds that obtained for the real data.

To finally compare the statistical evidence for an exponential distribution with that for a power-law distribution, we do the same analysis for fitting a power-law distribution. Since the input data are discrete, the MLE cannot be given in a closed form [11], we numerically maximize the log-likelihood

7 $$\begin{aligned} \mathcal {L}(\alpha ,{\hat{c}}_{\text {min}}) = -n({\hat{c}}_{\text {min}})\zeta (\alpha ,{\hat{c}}_{\text {min}}) - \alpha \sum _{j:\ c_j\ge {\hat{c}}_{\text {min}}}\ln c_j. \end{aligned}$$

A detailed comparison between fitting exponential and power-law distribution to the commenting data, including the log-likelihood test which directly compares the likelihood that the analyzed data has been drawn from the exponential or the power-law distribution, is presented in Sec. S2 in the SI.

## Supplementary Information

Below is the link to the electronic supplementary material.

Supplementary file1 (PDF 1445 kb)

## Data Availability

Upon publication of the manuscript, the BBC commenting data and scripts to reproduce the results presented here will be made available at https://github.com/8medom/Article-Impact.

## Footnotes

1 https://www.theverge.com/2020/11/24/21612728/facebook-news-feed-us-election-change-mainstream-news-misinformation.

2 https://www.nytimes.com/2017/12/20/insider/our-most-commented-on-articles-of-2017.html.

3 Strictly speaking, *P*(*c*) should be referred to as a geometric distribution as *c* is a discrete variable.

4 This normalization is also motivated by the observation that under negligible preferential attachment, $\varDelta c_i(t)/c_i$ is expected to accurately capture the aging function of the articles, as shown in the next section.

5 The minor difference with respect to Fig. 4 where the fitted timescale is 305 min is due to the use of wider bins in Fig. 6A to improve the statistics for high article age.

6 Data obtained from https://www.kaggle.com/aashita/nyt-comments.
