# Supplementary material for: The simple regularities in the dynamics of online news impact
Source: J Comput Soc Sci. 2021 Sep 12;5(1):629–46. doi: 10.1007/s42001-021-00140-w (PMC9114099; doi:10.1007/s42001-021-00140-w)
Supplement: Supplementary file 1 — Supplementary file1 (PDF 1445 kb) [file 42001_2021_140_MOESM1_ESM.pdf]

The simple regularities in the dynamics of online news impact
Supplementary Information

Matúš Medo, Manuel S. Mariani, Linyuan Lü

March 15, 2021

S1 Data description

S1.1 The BBC article discussion data

We collected a comprehensive dataset of sport news articles with discussions by periodically crawling the BBC Sport website (its front page and the pages dedicated to individual sports). In the time period from October 1, 2018 until June 30, 2019 (273 days), there were 3,087 article discussions open that received 852,400 comments from 67,527 users. The user median and mean number of comments are 2 and 12.6, respectively. The median and mean number of comments of a news article are 155 and 276, respectively. We measure the news article impact by the number of unique users who left a comment in its discussion. The median and mean article impact are 108 and 180, respectively.

Each comment is time-stamped with the time resolution of one minute. BBC typically closes discussions on the second midnight after the corresponding news article has been published; most of them are therefore open for 24–48 hours. There are two exceptions in the dataset: one discussion that has been open marginally longer than 48 hours and another discussion that was open for 13 days; it attracted only a few comments after day two, though.

S1.2 The NYT article discussion data

We further support our findings using the New York Times (NYT) commenting data obtained from https://www.kaggle.com/aashita/nyt-comments. Our NYT dataset comprises articles published in January–May 2017. At the NYT, it is possible to comment on a previously written comment (in fact, several levels of response are possible). To measure the article impact, we consider only the top-level comments; responses to comments are neglected as they are driven by the comments to which the responses are made. There are 2,801 articles and 649,794 comments from 75,118 users. The user median and mean number of comments are 1 and 6.0, respectively. The median and mean number of comments of a news article are 38 and 165, respectively. We measure the news article impact by the number of unique users who left a comment in its a discussion. The median and mean article impact are 37 and 143, respectively.

Each comment is time-stamped with the time resolution of one minute. Unlike the BBC data, article discussions at the NYT remain open for long time. Despite this, the article commenting activity decays quickly and the median time to reach 99% of the final comment count is approximately 26 hours (taking into account the hit articles whose comment count is above the 90th percentile).

Category	Articles	Mean comment count	Mean number of unique users (impact)
Football	1590	361	236
Rugby Union	439	219	130
Cricket	240	213	133
Tennis	162	157	108
Formula 1	139	338	209
Golf	123	117	85
Boxing	103	195	152
National	348	678	563
Learning	306	59	52
Magazine	262	77	72
Sports	213	48	45
Foreign	204	277	241
Games	192	31	29
Dining	166	40	39
Science	151	68	64
Upshot	146	124	115
Well	142	47	45
Metro	137	53	48
Business	109	269	239
Insider	105	28	27

Table S1: **Categories with at least 100 articles in the BBC data (top) and the NYT data (bottom).** Article categories are provided directly by both media outlets.

Rank	Impact	Category	News title
1	3538	football	Jose Mourinho: Manchester United sack manager
2	2241	football	Liverpool 4-0 Barcelona (4-3 agg): Jurgen Klopp's side complete extraordinary comeback
3	1931	football	Chris Hughton: Brighton sack manager after 17th-placed finish in Premier League
4	1874	football	Ajax 2-3 Tottenham (3-3 on aggregate - Spurs win on away goals): Lucas Moura scores dramatic winner
5	1697	football	Champions League: PSG 1-3 Man Utd (agg: 3-3)
6	1659	football	Manchester City 4-3 Tottenham Hotspur (4-4 agg): Spurs stun City on away goals in modern classic
7	1575	football	Liverpool, Tottenham, Chelsea and Arsenal fans criticise Uefa for final ticket numbers
8	1417	football	Ole Gunnar Solskjaer was the wrong choice as Man Utd manager - Jenas
9	1342	tennis	Andy Murray: Australian Open could be last tournament
10	1291	football	Liverpool beat Spurs 2-0 to win Champions League final in Madrid
1	3983	national	Trump Intensifies Criticism of F.B.I. and Journalists
2	3277	national	Trump Fires Comey Amid Russia Inquiry
3	3077	business	Man Is Dragged From a Full Jet, Stirring a Furor
4	2731	national	G.O.P. Revolt Sinks Bid to Void Health Law
5	2692	national	Judge Blocks Trump Order On Refugees
6	2618	foreign	Trump Is Said to Expose Ally's Secrets to Russians
7	2572	national	Britain Furious as Trump Pushes Claim of Spying
8	2388	national	Trump Was Told of Claims Russia Has Damaging Details on Him
9	2327	national	Trump Appealed to Comey to Halt Inquiry Into Aide
10	2304	national	Trump Fires Justice Chief Who Defied Him

Table S2: **Discussions with the highest impact as measured by the number of unique commenting users.** The BBC data (top) and the NYT data (bottom).

BBC:

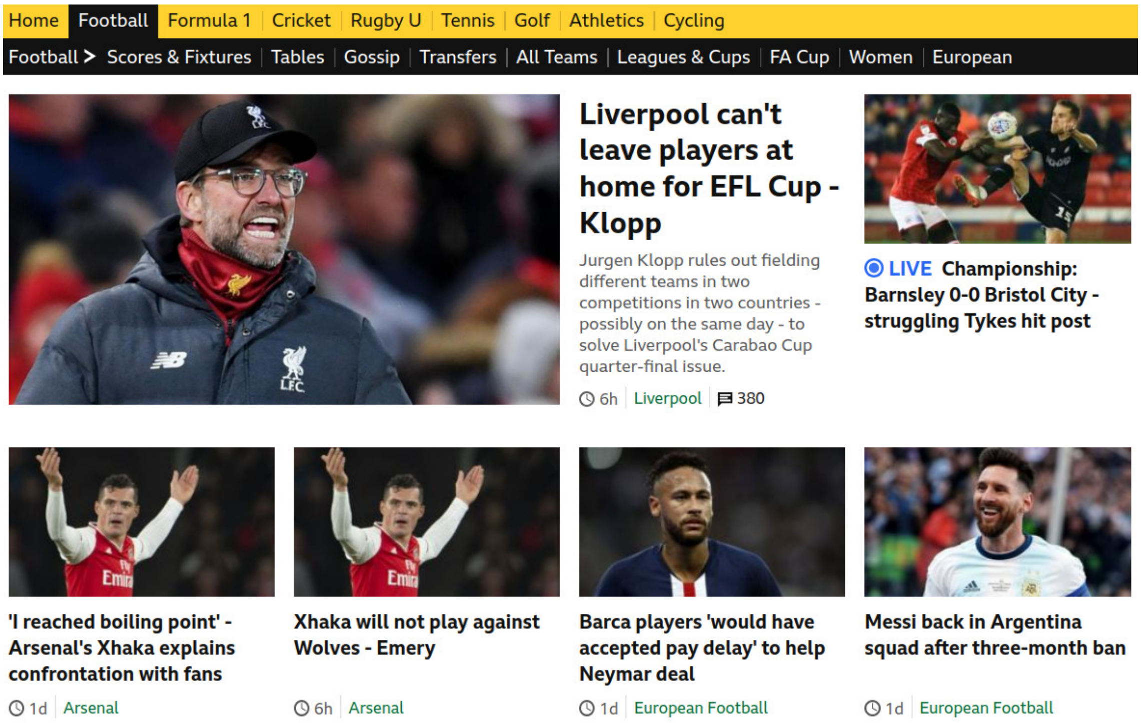

NYT:

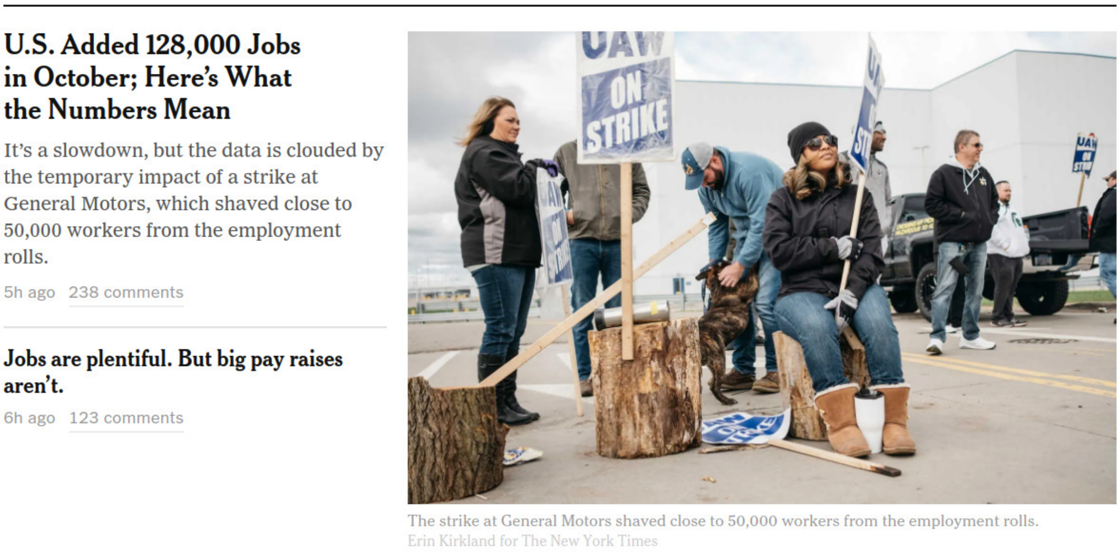

Figure S1: **Snapshots of the BBC website (top) and the NYT website (bottom).** Note that for articles with discussions, the number of comments is indicated for both BBC (icon with number 380 next to it; the other displayed articles do not have discussions) and NYT (labels "238 comments" and "123 comments").

S2 Fitting exponential distributions to article impact data

The primary fitting results for the BBC data are shown in Table S3 where article impact is measured by the number of unique users who comment on a news article. Since a minority of comments are discarded in this way, the fitting results are qualitatively similar when the total number of comments is used instead. Our fitting procedure follows the steps described in [1]: We choose the lower bound, $c_{\min}$, that minimizes the standard Kolmogorov-Smirnov statistic. The scaling parameter λ is then obtained by maximizing the data likelihood for the exponential model. The standard error of this estimate is then obtained by taking the standard deviation of scaling parameters estimated in bootstrap samples of the original data. Finally, the p-values characterizing the goodness-of-fit are obtained by comparing the corresponding Kolmogorov-Smirnov statistic measured on the real data with the Kolmogorov-Smirnov statistic measured on data drawn from the exponential distribution with the previously determined lower bound $c_{\min}$ (which directly influences the sample size represented by the number of article discussions that match or exceed $c_{\min}$) and the scaling parameter λ. Upon generating a large number of exponentially distributed samples, the p-value is the fraction of the samples that have a higher Kolmogorov-Smirnov statistic than the value found in the real data. A low p-value is thus an indication that the artificial exponentially-distributed samples match the fitted exponential distribution better then the real data. p-values above 0.10 are conventionally understood as an indication that the fit is good. Needless to say, the p-values are jointly influenced by the quality of fit and the sample size. As the sample size grows, the same deviation from the exponential distribution results in progressively lower p-values.

Category	N	O	$c_{\min}$	$f_{\min}$	KS	λ	p-value
Boxing	103	0	6	0.98	0.041	149 ± 15	0.97
Cricket	240	0	38	0.90	0.043	107 ± 8	0.58
Football	1,590	1	381	0.18	0.026	270 ± 17	0.96
Formula 1	139	0	42	0.94	0.052	180 ± 16	0.69
Golf	123	0	1	1.00	0.082	84 ± 10	0.13
Rugby-union	439	0	13	0.97	0.033	121 ± 7	0.49
Tennis	162	1	97	0.32	0.044	124 ± 17	1.00

Table S3: **Results of fitting exponential distributions to individual article categories: BBC data, article impact measured by the number of unique commenting users.** The displayed characteristics are: the number of articles with a discussion, N, the number of outliers, O, the determined lower bound of the exponential tail, $c_{\min}$, the fraction of articles that comprise the exponential tail, $f_{\min}$, the lowest Kolmogorov-Smirnov statistic, KS, the determined scaling parameter, λ, together with its standard error, and the p-value of the fit (p-values below some threshold, such as 0.10, would indicate that the exponential fit of the corresponding data is of low quality; for p-values above the threshold, the exponential distribution is not ruled out).

A single outlier—a news article with 3538 unique users contributing to the discussion—has been identified for the football category in the BBC data. When this news is included in the fitting procedure described above, we obtain $c_{\min} = 7$ and $\hat{\lambda} = 231$. The probability that the value of at least 3538 is observed in a single draw from the exponential distribution with these parameters is $p_0 \approx 2.3 \cdot 10^{-7}$. With 1577 independent draws from the distribution, corresponding to 1577 news that pass the impact threshold $c_{\min} = 7$, the probability that at least one of them has impact 3538 or more is $1-(1-p_0)^{1577} \approx 3.7 \cdot 10^{-3}$.

In other words, this single observation is very unlikely given the fitted exponential distribution which indicates that it is an outlier. The situation is similar in the tennis category where a single news with impact 1342 has probability $8.4 \cdot 10^{-3}$ to emerge for the fitted parameters $c_{\min} = 92$ and $\hat{\lambda} = 142$ (unlike Table S3, these results correspond to fitting all data including the outlier). In all other categories, the largest impact value has probability more than 0.01 to be observed.

Interestingly, the two identified outliers transcendent the sport boundaries which could help them reach the outstanding impact. The football category outlier is the news "Jose Mourinho: Manchester United sack manager" concerning a successful yet controversial coach and the tennis category outlier is the news "Andy Murray: Australian Open could be last tournament" concerning the potential end of career of the British most successful tennis player in the modern history.

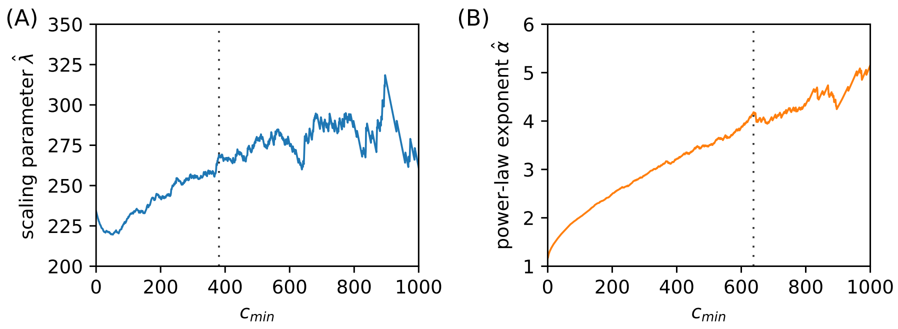

Figure S2: **The dependence of the parameter estimates on the fitting lower bound: a comparison between the exponential and power-law distribution, football category in the BBC data.** The exponential scaling parameter MLE estimate, $\hat{\lambda}$, varies little over a broad range of the fitting lower bounds, $c_{\min}$. By contrast, the power-law exponent MLE estimate, $\hat{\alpha}$, grows steadily and substantially with $c_{\min}$ which indicates that a power-law is not a good fit of the data. In both panels, the vertical dashed line shows the lower bound estimates which are obtained by minimizing the Kolmogorov-Smirnov statistic with respect to $c_{\min}$.

The same fitting analysis using power-law distributions shows that they yield worse fit of the data than exponential distributions. This can be visually appreciated in Figure S2 which shows the dependence of estimated parameters on the fitting lower bound, $c_{\min}$. While the estimated scaling parameter of the exponential distribution, $\hat{\lambda}$, varies in the narrow range $[225, 290]$, estimated exponent of the power-law distribution, $\hat{\alpha}$, continually grows with $c_{\min}$ from an extremely low value of 1.2 to more than five and shows no plateau. The higher stability of $\hat{\lambda}$ as compared to $\hat{\alpha}$ is a sign that the exponential fits are more robust (less sensitive to the choice of $c_{\min}$, in particular). Finally, the smallest Kolmogorov-Smirnov statistic for a power-law fit of the most populated football category news yields $c_{\min} = 638$, so the power-law spans over less than one order of magnitude unlike other broadly distributed datasets [1].

Table S4 further reports detailed results of fitting with power-law distributions. We see that the lower bounds obtained for power-law fits are substantially higher than the corresponding values for exponential fits, which shows that exponential distributions fit greater portions of respective datasets. The Kolmogorov-Smirnov statistic values obtained with exponential fits are lower than those obtained with power-law fits, indicating better match between the data and the fitted distributions. We conclude the assessment of power-

	Power-law fit				Likelihood ratio test	
Category	$c_{\min}$	$f_{\min}$	KS	$\hat{\alpha}$	LR	p-value
Boxing	233	0.24	0.054	3.75	13.5	0
Cricket	147	0.32	0.057	3.11	38.2	0
Football	638	0.07	0.068	4.18	64.2	0
Formula1	141	0.58	0.081	2.54	29.4	0
Golf	83	0.33	0.059	2.64	11.3	0
Rugby union	279	0.12	0.065	4.03	36.4	0
Tennis	189	0.17	0.076	3.15	14.1	0

Table S4: **Results of fitting power-law distributions to individual article categories and the likelihood ratio tests, BBC data.** We measure article impact by the number of unique users who have commented on it and use the standard Kolmogorov-Smirnov statistic for the fitting analysis. We show here the results of the power-law fitting (the estimated lower bound, the fraction of article discussions that comprise the identified power-law tail, and the estimated power-law exponent) as well as the likelihood test results (the normalized log likelihood ratio, LR, that measures the difference in how well the fits agree with the data and the corresponding p-values that estimates how likely it is to find LR as high or higher by chance).

law and exponential fits by a direct comparison between the exponential and power-law distribution in terms of how well they fit the data (to allow for a fair comparison, we do not exclude any outliers here as that would put the power-law hypothesis in a disadvantage). This can be done using the log-likelihood test described in [1]. As can be seen in Table , the normalized log likelihood ratio [4], LR, favors the exponential distribution for all analyzed news categories and the corresponding p-values show that the obtained LR values are highly significant (p-values 10^{-28} and less).

Finally, Figure S3 visually compares exponential and power-law fit for all news categories. While two categories—Golf and Rugby Union—display an excess of high-impact news, those high-impact news are fewer than ten in both cases, so they do not suffice to turn around the likelihood ratio test. The piece-wise linear shape of the distributions for Football and Tennis suggests that their fits could be improved by using a combination of two exponentials with different scale parameters, corresponding to two different classes of news mixed in the same category. Interestingly, the same pattern can be seen also for some NYT news categories in Figure S5 (categories Dining and Foreign, for example). This suggests the hypothesis that while a homogeneous news category is well fitted with a single exponential, a mixture of two exponential distributions may be more appropriate for a heterogeneous news category. Further work is needed to explore such possible fine structure of news categories.

We applied the same steps to the NYT data where the article impact is measured by the number of unique commenting users at the top commenting level (see Section S1 for details), focusing on the 13 news categories with at least 100 news. Table S5 summarizes the results of exponential fitting, showing that the exponential distribution again cannot be ruled out for any news category, albeit now there are two categories—Learning and Magazine—where only a small fraction of news belong to the exponential tail and the best Kolmogorov-Smirnov statistic for Learning news is high (0.148). Figure S4 illustrates the stability of the scaling parameter estimate over a broad range of the lower fitting bound, $c_{\min}$, whereas the estimated power-law exponents substantially grows with $c_{\min}$.

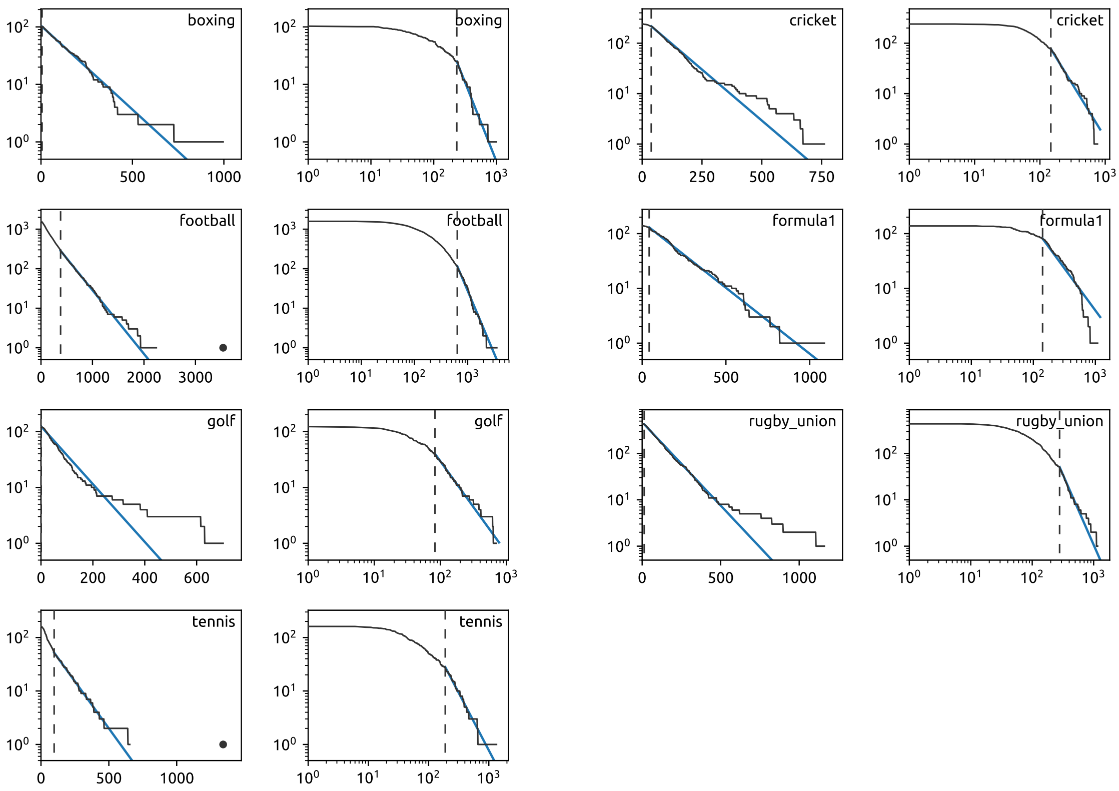

Figure S3: **Visualizations of MLE fits for individual news categories, BBC data.** Each news category is displayed twice: in the log-linear scale (left) with the exponential fit and in the log-log scale (right) with the power-law fit. The vertical dashed lines mark the estimated lower fitting bounds.

Category	N	O	c_{min}	f_{min}	KS	λ	p-value
Business	109	1	0	0.99	0.064	213 ± 23	0.52
Dining	166	0	31	0.36	0.068	54 ± 6	0.80
Foreign	204	0	214	0.31	0.060	396 ± 52	0.91
Games	192	0	35	0.34	0.056	6.4 ± 0.9	0.95
Insider	105	0	2	0.97	0.081	26 ± 3	0.22
Learning	306	0	72	0.09	0.148	352 ± 72	0.27
Magazine	262	0	118	0.13	0.071	217 ± 35	0.97
Metro	137	0	47	0.26	0.083	96 ± 17	0.86
National	348	0	28	0.97	0.037	567 ± 32	0.53
Science	151	1	0	0.99	0.049	60 ± 4	0.63
Sports	213	0	49	0.25	0.081	78 ± 12	0.65
Upshot	146	1	34	0.84	0.062	90 ± 9	0.47
Well	142	0	3	0.99	0.050	43 ± 3	0.65

Table S5: **Results of fitting exponential distributions to article categories: NYT data, article impact measured by the number of unique users at the top commenting level.** Notation as in Table S3.

Finally, Table S6 summarizes results of power-law fits and likelihood ratio tests for individual article categories. Category Learning is confirmed here as the only one for which the exponential fit is worse than the power-law fit (normalized log likelihood ratio, LR,

is significantly negative; see Sec. S2.1 for more information on this category). For the remaining 12 categories, exponential fits are much more apt than power-law fits. Figure S5 visually compares exponential and power-law fit for all news categories.

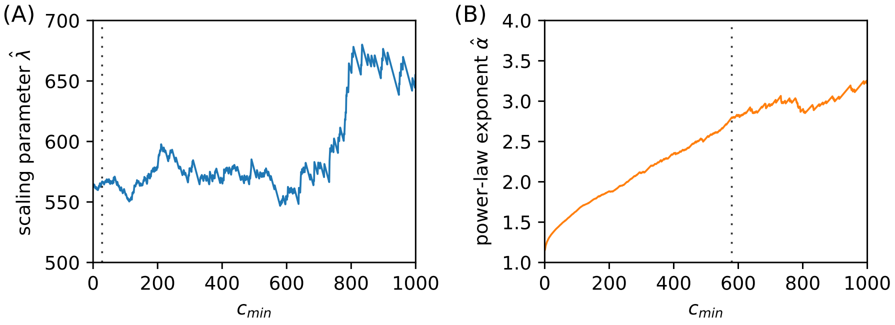

Figure S4: **The dependence of the parameter estimates on the fitting lower bound: a comparison between the exponential and power-law distribution, National category in the NYT data.** The exponential scaling parameter MLE estimate, $\hat{\lambda}$, varies little over a broad range of the fitting lower bounds, $c_{\min}$. By contrast, the power-law exponent MLE estimate, $\hat{\alpha}$, grows steadily and substantially with $c_{\min}$ which indicates that a power-law is not a good fit of the data. In both panels, the vertical dashed line shows the lower bound estimates which are obtained by minimizing the Kolmogorov-Smirnov statistic with respect to $c_{\min}$.

S2.1 Specifics of the category "Learning" in the NYT data

The Learning category has a particularly uneven impact distribution: While the median impact over its 306 news is only 7, there are 23 news with impact above 100 and the top impact is 2,344. Nevertheless, it turns out that the Learning category actually does not contain news but a mixed bag of content. Among the 23 news with impact above 100, there are 17 contest articles "What's Going On in This Picture?" (published on a weekly basis) with the median impact of 505 and the top impact article is yet another contest.[1] By contrast, little impact Learning articles are "teaching and learning resources based on New York Times journalism" made available under the name "Learning Network".[2]

[1] https://www.nytimes.com/2017/04/05/learning/our-eighth-annual-found-poem-student-contest.html

[2] See https://www.nytimes.com/2017/04/18/learning/what-does-a-presidents-choice-of-pet-or-choice-not-to-hav html for an example.

	Power-law fit				Likelihood ratio test	
Category	$c_{\min}$	$f_{\min}$	KS	$\hat{\alpha}$	LR	p-value
Business	224	0.39	0.086	2.82	9.7	0
Dining	13	0.71	0.095	1.92	13.2	0
Foreign	521	0.15	0.100	3.11	6.2	0
Games	33	0.44	0.063	6.48	31.5	0
Insider	8	0.85	0.101	1.92	10.5	0
Learning	16	0.34	0.074	1.75	−4.9	0
Magazine	57	0.31	0.070	2.15	4.8	0
Metro	7	0.83	0.095	1.66	4.9	0
National	580	0.37	0.081	2.80	24.4	0
Science	108	0.21	0.115	3.56	12.0	0
Sports	69	0.21	0.069	2.68	8.1	0
Upshot	63	0.64	0.084	2.36	22.2	0
Well	85	0.16	0.087	3.52	14.3	0

Table S6: **Results of fitting power-law distributions to individual article categories and the likelihood ratio tests, NYT data.** We measure article impact by the number of unique users who have commented on it and use the standard Kolmogorov-Smirnov statistic for the fitting analysis. We show here the results of the power-law fitting (the estimated lower bound, the fraction of article discussions that comprise the identified power-law tail, and the estimated power-law exponent) as well as the likelihood test results (the normalized log likelihood ratio, LR, that measures the difference in how well the fits agree with the data and the corresponding p-values that estimates how likely it is to find LR as high or higher by chance).

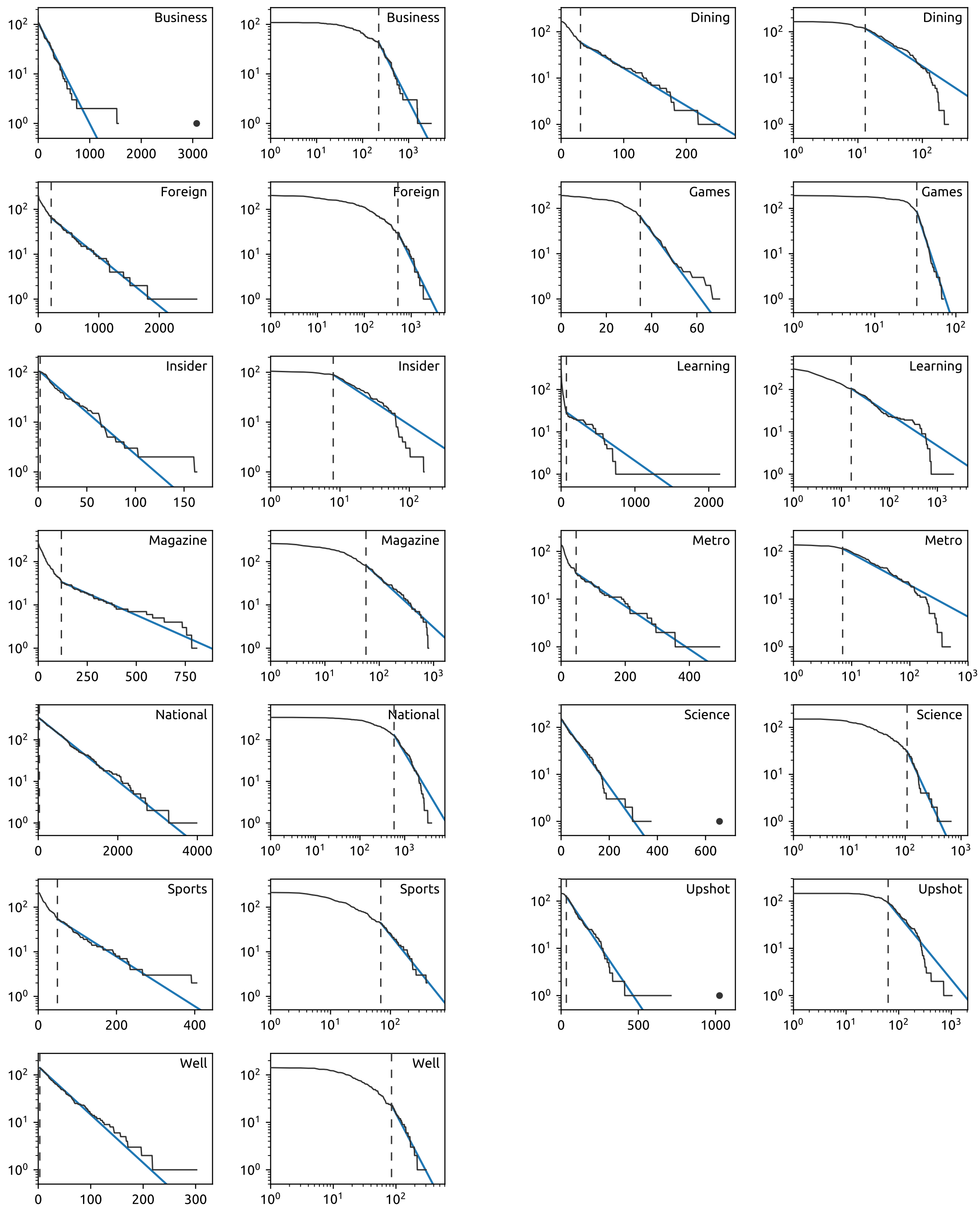

Figure S5: **Visualizations of MLE fits for individual news categories, NYT data.** Each news category is displayed twice: in the log-linear scale (left) with the exponential fit and in the log-log scale (right) with the power-law fit. The vertical dashed lines mark the estimated lower fitting bounds.

S3 Elementary analysis of the system's dynamics

We present here basic characteristics of the commenting dynamics in the BBC and the NYT data: the daily and hourly profiles of user activity and the distributions of user activity (Figures S6–S8).

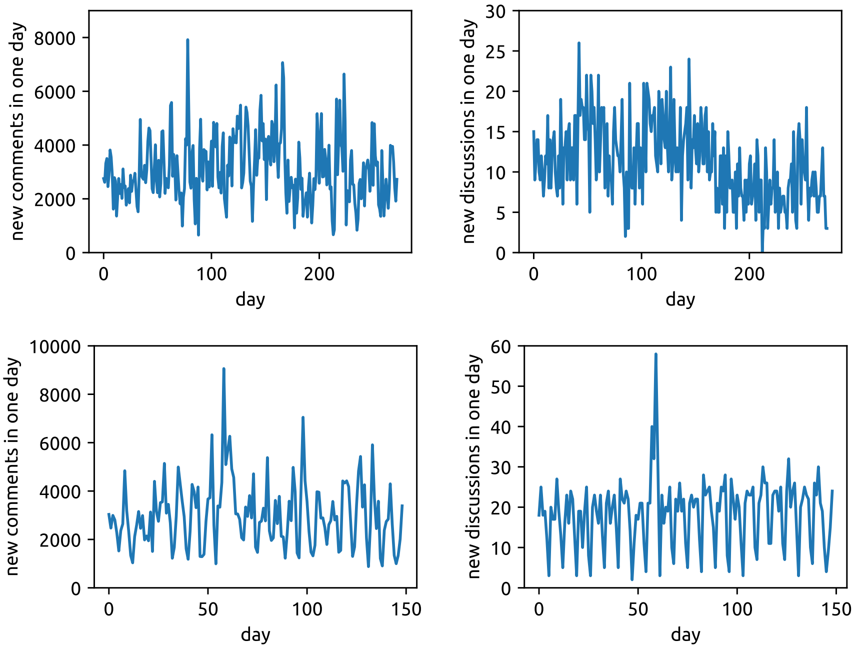

Figure S6: **Variations of the daily commenting activity.**
(Top row, BBC data) There are 3,109 new comments a day on average, the standard deviation is 1,215. There are 11.3 new article discussions a day on average, the standard deviation is 4.8.
(Bottom row, NYT data) There are 3,007 new comments a day on average, the standard deviation is 1,347. There are 18.4 new article discussions a day on average, the standard deviation is 8.2.

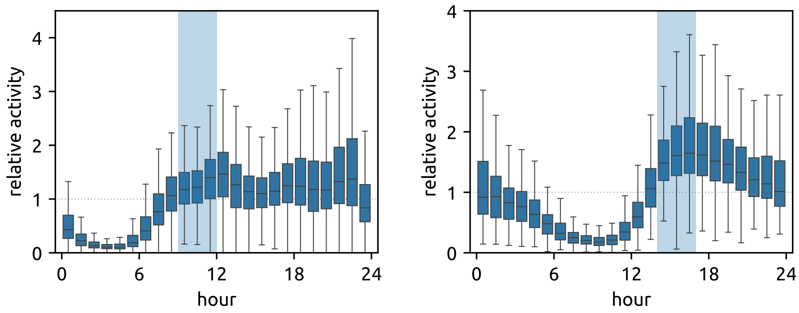

Figure S7: **Variations of the normalized commenting activity during the day.** The value of one corresponds to 1/24 of the day's comments arriving in a given hour.
(Left, BBC data) The activity is low from 1am to 6am and relatively constant between 8am and midnight. The shaded area shows the time of day that we use for the analysis of the commenting dynamics: These articles have a long period of approximately uniform website activity before the night arrives and the activity drops.
(Right, NYT data) Due to the time difference, the commenting activity is lower between 5am and noon. The shaded area again shows the "morning articles" that are used for the analysis of new dynamics.

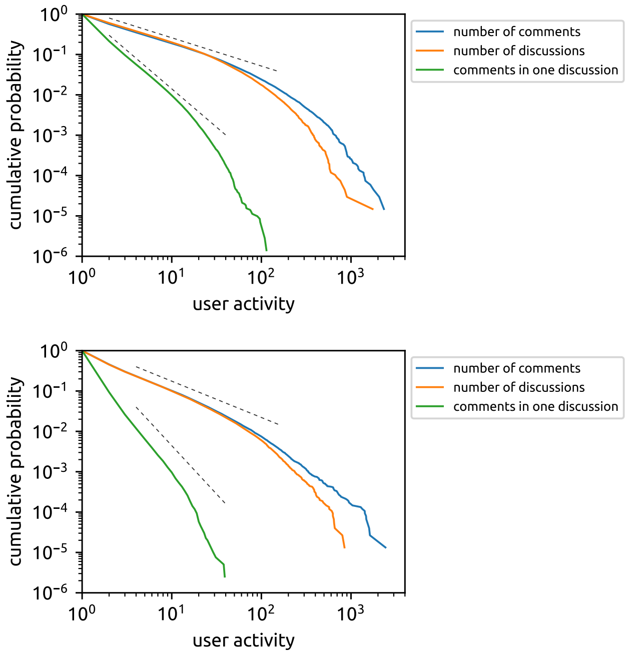

Figure S8: **The distributions of various measures of user activity.**
(Top, BBC data) The indicative dashed lines have the slopes of 0.7 and 1.9, respectively.
(Bottom, NYT data) The indicative dashed lines have the slopes of 0.9 and 2.4, respectively.
All three distributions have an initial power-law part with a cut-off at higher activity values.

S4 Preferential attachment in measurements and models

Figure 2A in the main text shows that preferential attachment is extremely weak in the BBC data with the linear fit for $c_i(t) < 800$ in the form $\Delta c_i(t) \sim 1 + c_i(t)/220$ followed by saturation for higher $c_i(t)$ values. We add that a similar weak dependence of $\Delta c_i(t)$ on $c_i(t)$ can be observed in synthetic data where preferential attachment is absent. To demonstrate this, we use the model described in Section S5 which produces a synthetic dataset whose basic properties are similar to those of the BBC dataset. The measurement of preferential attachmed identical with the one used in the main text yields similar results as those in Figure 3A (see Figure S9) and a similar fit $\Delta c_i(t) \sim 1 + c_i(t)/336$. The observed weak growth of $\Delta c_i(t)$ with $c_i(t)$ is thus an artifact of the commenting dynamics produced by the model. In particular, articles with high final degree (comment count) initially increase their $c_i(t)$ fast, so their high $\Delta c_i(t)$ values contribute to the initial low $c_i(t)$ bins for a short time. By contrast, articles with low final degree remain during their whole lifetime in low $c_i(t)$ bins where they contribute with their low $\Delta c_i(t)$ values and hence cause the average $\Delta c_i(t)$ values to be lower in low $c_i(t)$ bins that they are in high $c_i(t)$ bins.

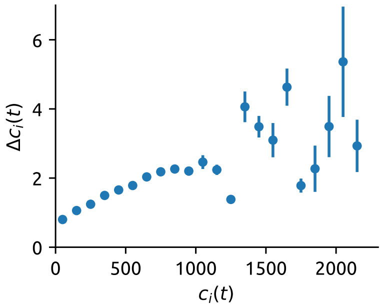

Figure S9: **Measuring preferential attachment in synthetic data without preferential attachment.** We use here the synthetic dataset produced using the model described in Section S5 without preferential attachment. The fit up to the comment count 800 yields the slowly-growing dependence proportional to $1 + c_i(t)/336$. Since there is no preferential attachment, the observed weak dependence of $\Delta c_i(t)$ on $c_i(t)$ is an artifact of the model's dynamics.

We claim in the main text that preferential attachment with saturation fails to produce a power-law degree distribution from exponentially distributed fitness values. This can be illustrated using the synthetic data model from Section S5 where the rate at which an article receives new comments (number of new comments per minute) at time t is assumed to be

$$\eta_i F[c_i(t)] D(t) \tag{1}$$

where η_i is the fitness of article i, $F[c_i(t)]$ is the degree-dependence term depending on the current number of comments, $c_i(t)$, and $D(t)$ is an aging term. We assume that article fitness is exponentially distributed, $\rho(\eta) = \exp(-\eta/\lambda)/\lambda$ for $\eta \in [0, \infty)$. The distribution's scale, λ, determines the resulting average comment count. Motivated by Figure 4 in the main text, we assume exponential aging, $D(t) = \exp[-(t - t_i)/\Theta]$ where t_i is the appearance time of article i and Θ is the aging timescale. For the degree-dependence term, we use three different choices. Firstly, degree-independent growth given by $F(c) = 1$. As shown in Sec. S5 and confirmed in Figure S11, the degree distribution is then exponential. Secondly, standard preferential attachment given by $F(c) = 1 + c$ (the addition of one is necessary as the initial comment count of all articles is zero). As shown in [3] and confirmed by

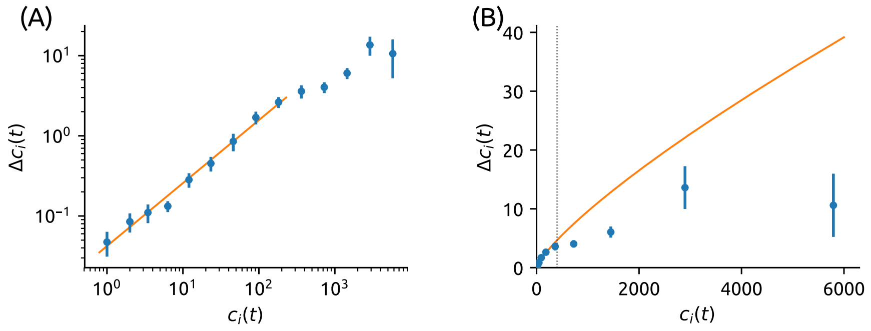

Figure S10: **Non-linear preferential attachment and its saturation in the NYT data.** To study non-linear preferential attachment, we use logarithmic bins on the x-axis and log-log scale to plot the same measurement of preferential attachment as in Figure 3B in the main text. (A) The linear fit of the first nine bins (the last bin includes degree values up to 256) has the slope of 0.79. (B) As panel (A) but with linear axes. The saturation of preferential attachment at, approximately, degree 400 (marked with the vertical dotted line) is well visible here.

the results in Figure S11, exponentially distributed fitness combined with preferential attachment produces a power-law degree distribution. Finally, preferential attachment with saturation given by $F(c) = 1 + c$ for $c < 100$ and $F(c) = 100$ for $c \geq 100$. As shown in Figure S11, the degree distribution then appears to be a power law (straight in the log-log scale) until $c \approx 100$ and then follows a clear exponential course for larger c. The importance of the saturation threshold is natural as the emergence of a power-law degree distribution is a direct consequence of the interplay between the exponential fitness distribution and preferential attachment. Since preferential attachment is absent above the saturation threshold, an exponential degree distribution emerges for large comment counts in the same way as it emerges for all comment counts when preferential attachment is absent entirely [$i.e.$, when $F(c) = 1$].

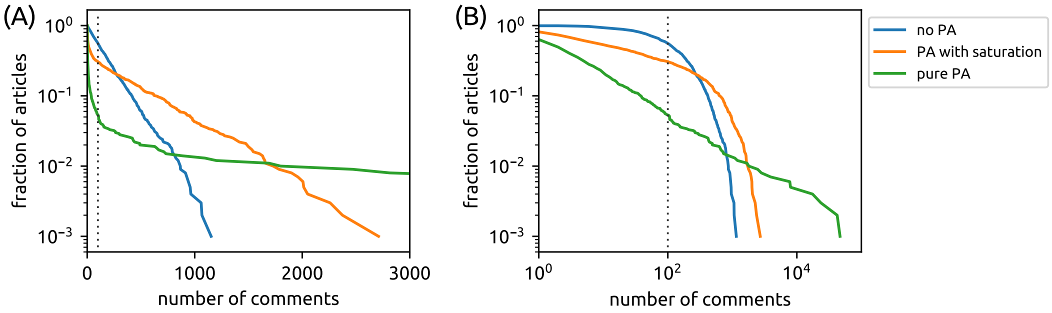

Figure S11: **Degree distributions for various variants of preferential attachment.** The two panels use a log-linear scale (A) and a log-log scale (B), respectively. Degree distributions of networks where the rates of node degree increase are $a\eta_i F(c_i)\exp(-t/\Theta)$, $\Theta = 240\,\mathrm{min}$. Node fitness is drawn from the exponential distribution, $\rho(\eta) = \exp(-\eta/\lambda)/\lambda$ for $\eta \in [0, \infty)$. We compare no preferential attachment [$F(c) = 1$, $\lambda = 0.75$], standard preferential attachment [$F(c) = 1 + c$, $\lambda = 0.007$], and preferential attachment with saturation [$F(c) = 1 + c$ for $c < 100$ and $F(c) = 100$ for $c \geq 100$, $\lambda = 0.018$]. The proportionality rates a are chosen so that the average degree is similar, approximately 180, in all three model variants. The vertical dotted lines mark the saturation threshold $c = 100$.

S5 Modeling the dynamics of news impact

Based on the results presented in the main text, we see that the article commenting dynamics is influenced by two principal factors:

1. *Absence of preferential attachment,*
2. *Exponential aging,*
3. *Overall user activity at the website.*

We assume for the moment that the overall user activity remains stable during the modeling period. A generalized model which accounts for varying user activity, as discussed in the last section of the main text, is presented in Eq. (4) in the main text. We further assume that exogeneous factors such as the article position on the BBC Sport website (on the front page or not, at the top of a page or not, and so forth—see Figure S1 or https://www.bbc.com/sport for examples). While article position might seem like an important factor, Figure S12 shows that commenting dynamics of high-impact articles does not display abrupt changes that could correspond to changes of each article's position. We thus neglect article position at the website which, in any case, is not contained in the collected data.

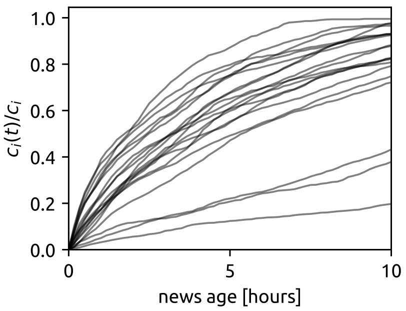

Figure S12: **The degree trajectories of the 20 highest impact articles in the BBC data.** To suppress the time-of-the-day effects during the displayed 10-hour period, we select only the articles that appear in the morning (as in Figure 4 and 5 in the main text). Changing an article's position at the website has the potential to influence its exposure to the users and, in turn, the rate at which the article receives new comments. The displayed individual trajectories do not exhibit substantial changes of the commenting rates which indicates that the effect of changing the articles' positions is minor and can be neglected in the scope of our analysis of commenting dynamics.

The factors 1 and 2 in the list above correspond to the expected number of new comments in the form

$$\overline{\Delta c_i(t)}/\Delta t = \eta_i \exp[-(t - t_i)/\Theta] \tag{2}$$

where $c_i(t)$ is the number of comments of article i at time t, η_i is the fitness of article i which reflects how attractive it is to the users, t_i is the time when the news article was published (and its discussion opened, $c_i(t_i) = 0$), and finally Θ_i is the article's aging timescale. The actual number of new comments is drawn from the Poisson distribution with mean given by Eq. (2). This choice is motivated by independent actions of the users. In Figure S13, we illustrate that the Poisson distribution is indeed a good approximation by studying the number of new comments that article discussions receive in two consecutive time windows. The results are grouped by the total number of comments an article discussion receives in

the two time windows and we plot the difference of the number of comments between the two time windows. This distribution is then compared with the distribution of differences observed when the comments counts are drawn from a Poisson distribution. As can be seen in the figure, the empirical distributions are indeed similar with the distributions produced by Poisson-distributed comment counts.

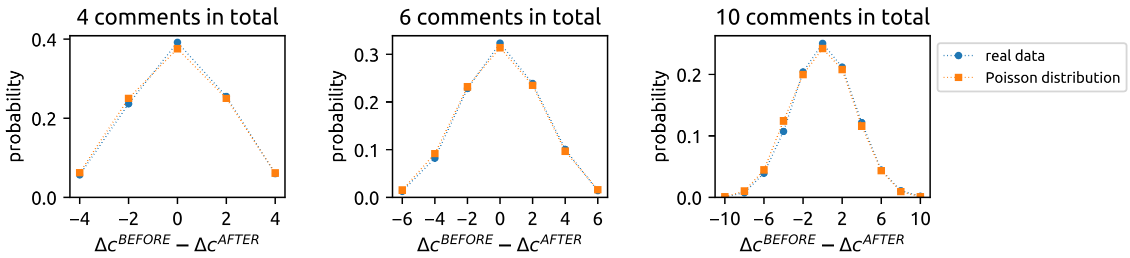

Figure S13: **Probing the comment count randomness in the BBC data.** Using many pairs of consecutive time windows of length $\Delta T = 10\,\text{min}$ each, we collected comment count increase values Δc^{BEFORE} and Δc^{AFTER} for a large number of articles. The histograms compare the empirical distributions of $\Delta c^{BEFORE} - \Delta c^{AFTER}$ with the distributions obtained for Poisson-distributed comment counts. For an easier interpretation of the results, the histograms are plotted separately for observations where $\Delta c^{BEFORE} + \Delta c^{AFTER}$ is 4, 6, and 10, respectively.

The discrete model given by Eq. (2) combined with draws from the Poisson distribution can be replaced with the continuous modeling using the differential equation

$$\frac{\mathrm{d}\overline{c_i(t)}}{\mathrm{d}t} = \eta_i \exp[-(t - t_i)/\Theta_i] \tag{3}$$

with the initial condition $c_i(t_i) = 0$. This is referred to as the Fitness-Aging (FA) model in the main text. A similar model was proposed in [2] which explores the relation between fitness and preferential attachment. Eq. (3) is easy to solve and yields

$$\overline{c_i(t)} = \eta_i \Theta_i \biggl(1 - \exp\bigl[-(t - t_i)/\Theta_i\bigr]\biggr). \tag{4}$$

with the limit value $\overline{c_i} = \eta_i \Theta_i$. In the absence of preferential attachment, the final impact of an article is directly proportional to its fitness and aging timescale. Although we found sub-linear preferential attachment in the NYT data, Figure 5B in the main text shows that Eq. (4) mathces the impact dynamics in the NYT data well despite being derived assuming that preferential attachment is absent.

The generalized form of Eq. (3) is

$$\frac{\mathrm{d}\overline{c_i(t)}}{\mathrm{d}t} = \eta_i F[c_i(t)] \exp[-(t - t_i)/\Theta_i] \tag{5}$$

where $F[c_i(t)]$ is a preferential attachment term. Standard preferential attachment is represented with $F[c_i(t)] = C + c_i(t)$ where C is an additive term which is necessary as all articles have initially $c_i(t_i) = 0$. Non-linear preferential attachment is represented with, for example, $F[c_i(t)] = C + c_i(t)^{\beta}$. Preferential attachment with saturation is represented with a piece-wise function of $c_i(t)$. The resulting distribution of article impact for various choices of $F[c_i(t)]$ is studied in Section S4 where we show that when the preferential

attachment dependence in $F[c_i(t)]$ has a cut-off, the tail of the impact distribution has the same distribution as $\eta_i \Theta_i$. This contrasts with the results presented in [3] where the authors show that exponentially distributed fitness combined with preferential attachment gives rise to a power-law degree distribution.

S6 Estimating individual aging timescales

Note that we assume an individual aging timescale, Θ_i, for each article. One can ask whether this generalization is really necessary, if universal aging timescale Θ would not yield a similar level of agreement between the model and the empirical data. We illustrate this in Figures S14 and S15 below where the left panels use real time on the x axes and the middle panels use rescaled time $(t - t_i)/\Theta_i$ on the x axes. The aging timescales are estimated by minimizing the Kolmogorov-Smirnov statistic between the real course of $c_i(t)$ and the theoretically expected curve given by Eq. (4). For the BBC data, the lowest mean KS is achieved with the universal timescale of 300 minutes which agrees with the timescale obtained by fitting the aging curve in Figure 4A in the main text; the mean KS static is then 0.18 and 9 articles (out of the 39 used to obtain the figure) have the KS statistic below 0.1. With individually estimated timescales, the mean Kolmogorov-Smirnov statistic reduces by the factor of two to 0.08 and 34 articles have the KS statistic below 0.1. For the NYT data, the lowest mean KS is achieved with the universal timescale of 330 minutes; the mean KS is then 0.27 and 5 article discussions out of 46 have the KS statistic below 0.1 (the values are 0.29 and 4 for the timescale 230 obtained by fitting the aging curve in Figure 4B in the main text). With individually estimated timescales, the mean KS reduces to 0.12 and 30 articles have the KS statistic below 0.1. The improvement achieved by using Eq. (5) which includes preferential attachment is minor both in terms of the mean KS as well as the number of articles with KS below 0.1. To summarize, the commenting dynamics described by Eq. (4) fits the empirical data well even when some limited effects of preferential attachment can be observed in the NYT data (as shown in Figure 3B in the main text).

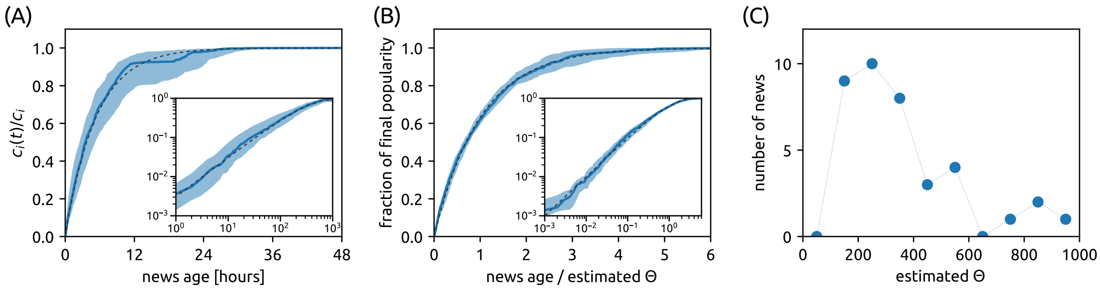

Figure S14: **Commenting dynamics in the BBC data.** (A) The dynamics of article impact in the real time. The solid line shows the median fraction of the final popularity at given article age; the shaded region shows the 20th–80th percentile range of the observed popularity fraction values. The dashed line corresponds to Eq. (4) with the aging timescale $\Theta = 305\,\text{min}$. (B) As panel (A) but time is rescaled with the individual aging timescale Θ for each article (same Figure 5A in the main text). The inset focuses on the early time after the article appearance using a log-log scale. (C) The distribution of the individual aging timescales among the articles. As in Figure 5 in the main text, we include morning hit articles in the analysis here.

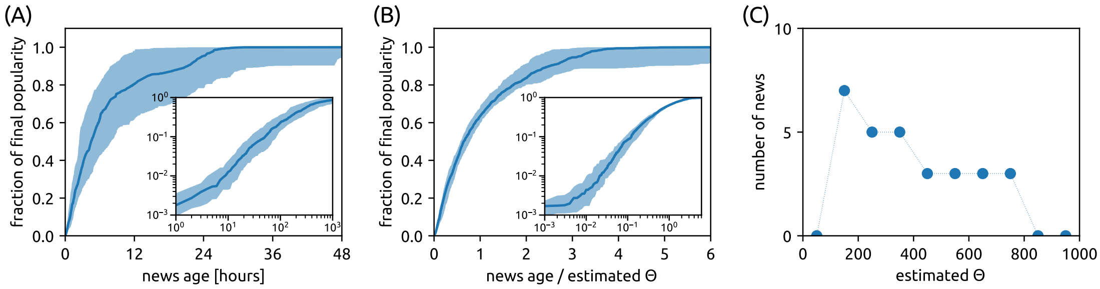

Figure S15: **Commenting dynamics in the NYT data.** All panels as in Figure S14.

S7 Interplay between exponential aging and circadian user activity patterns for the NYT data

Since the overall user activity varies in the NYT data in a similar way as it does in the BBC data, its impact on article impact dynamics can be studied analogously to the section "Interplay between exponential aging and circadian user activity patterns" in the main text where results for the BBC data are presented. While the aging timescale of morning articles in the NYT data is $\Theta_A = 230\,\mathrm{min}$ (see Figure 4B in the main text), Figure S16A shows that the aging timescale is significantly shorter, 126 minutes, for evening articles. Figure S16B shows that the overall user activity decays in the with the fitted timescale of $\Theta_U = 200\,\mathrm{min}$. Using Eq. (5) from the main text, the corresponding joint timescale is

$$1/\Theta_J = 1/(230\,\mathrm{min}) + 1/(200\,\mathrm{min}) \quad \Longrightarrow \quad \Theta_J = 107\,\mathrm{min}$$

which is close to the actual fitted aging time scale of evening articles, 126 minutes.

Note that the NYT data are less suitable for this analysis than the BBC data for two main reasons. Firstly, evening user activity decay is piece-wise with the first slower part between 11 pm and 4 am and the second faster part between 4 am and 9 am (we use GMT). This is probably due to the large geographical size of USA which mainly covers four time zones (from the east coast to the west coast). In our analysis, we use the more pronounced second part when, however, the overall user activity is low which makes our statistical estimates more noisy. Secondly, as many articles appear on BBC Sport in the evening, Figure 6B in the main text is built on data from 56 evening hit articles. By contrast, the analogous Figure S16A uses only 13 NYT hit articles that appear in the evening which makes statistical estimates less precise. Thirdly, daily variations are greater in the BBC data (as measured by the ratio between the maximal and minimal user activity during the day, for example) which makes this dataset more suitable for analyzing the impact of daily variations on the dynamics of article impact.

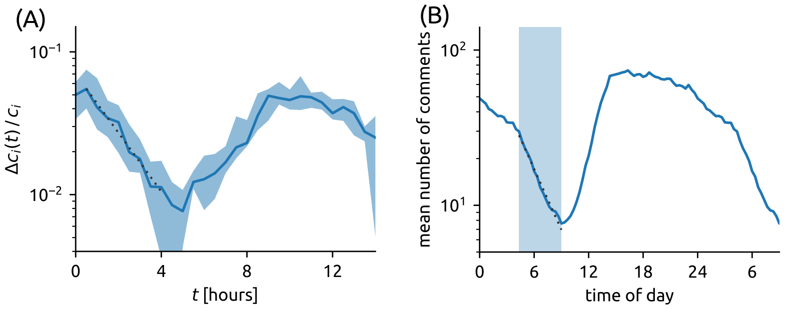

Figure S16: **Interplay between exponential aging and circadian user activity patterns in the NYT data.** (A) The number of new comments of an article, $\Delta c_i(t, \Delta t)$, normalized by the final number of comments, c_i, as a function of its age, t, for evening hit articles (published between 3 am and 6 am GMT). The dotted line indicates the linear fit for age 30–240 minutes (fitted timescale 126 minutes). The age bin size here is 30 minutes to achieve better statistics for high article age. (B) The variation of the mean number of comments in 20 minute intervals during the day in the NYT data. Fitted timescale of the exponential decay between 4 am and 9 am GMT is 200 minutes.

References

[1] Aaron Clauset, Cosma Rohilla Shalizi, and Mark EJ Newman. Power-law distributions in empirical data. *SIAM Review*, 51(4):661–703, 2009.

[2] Michael Golosovsky. Mechanisms of complex network growth: Synthesis of the preferential attachment and fitness models. *Physical Review E*, 97(6):062310, 2018.

[3] Matúš Medo, Giulio Cimini, and Stanislao Gualdi. Temporal effects in the growth of networks. *Physical Review Letters*, 107(23):238701, 2011.

[4] Quang H Vuong. Likelihood ratio tests for model selection and non-nested hypotheses. *Econometrica: Journal of the Econometric Society*, pages 307–333, 1989.
